# ATRX and Its Prognostic Significance in Soft Tissue Sarcoma

**DOI:** 10.1155/2024/4001796

**Published:** 2024-05-06

**Authors:** Mark M. Cullen, Warren Floyd, Bobby Dow, Beatrice Schleupner, Brian E. Brigman, Julia D. Visgauss, Diana M. Cardona, Jason A. Somarelli, William C. Eward

**Affiliations:** ^1^Department of Orthopaedic Surgery, Duke University Medical Center, Durham, NC, USA; ^2^Department of Radiation Oncology, The University of Texas MD Anderson Cancer Center, Houston, TX, USA; ^3^Texas A&M University Health Center College of Medicine, Bryan, TX, USA; ^4^Duke University, Durham, NC, USA; ^5^Duke Cancer Institute, Duke University Medical Center, Durham, NC, USA; ^6^Department of Pathology, Duke University Health System, Durham, NC, USA; ^7^Department of Medicine, Duke University Medical Center, Durham, NC, USA

## Abstract

**Purpose:**

Recently, the association between ATRX and a more aggressive sarcoma phenotype has been shown. We performed a retrospective study of sarcomas from an individual institution to evaluate ATRX as a prognosticator in soft tissue sarcoma. *Experimental Design*. 128 sarcomas were collected from a single institution and stained for ATRX. The prognostic significance of these markers was evaluated in a smaller cohort of primary soft tissue sarcomas (*n* = 68). Kaplan–Meier curves were created for univariate analysis, and Cox regression was utilized for multivariate analysis.

**Results:**

High expression of ATRX was found to be a positive prognostic indicator for overall survival and metastasis-free survival in our group of soft tissue sarcomas both in univariate analysis and multivariate analysis (HR: 0.38 (0.17–0.85), *P*=0.02 and HR: 0.49 (0.24–0.99), *P*=0.05, respectively).

**Conclusions:**

High expression of ATRX is a positive prognostic indicator of overall survival and metastasis-free survival in patients with STS. This is consistent with studies in osteosarcoma, which indicate possible mechanisms through which loss of ATRX leads to more aggressive phenotypes. Future prospective clinical studies are required to validate the prognostic significance of these findings.

## 1. Introduction

Soft tissue sarcomas (STS) are a rare group of malignant neoplasms arising from mesenchymal origin and encompassing over 100 different histological subtypes [1]. Survival of high grade soft tissue sarcomas has been cited to be approximately 50%, at 5 years without metastatic disease and 20% with metastatic disease [2]. With the advancement in molecular profiling and next-generation sequencing techniques, STS can be classified into unique subgroups [3], and such groupings may provide insight into prognostic or therapeutic insights for these patients. While the genomic underpinnings of STS are complex, three genes are consistently mutated in STS: TP53, RB1, and ATRX [4, 5]. Although TP53 [6–8] and RB1 [9] are well-known tumor suppressors across many cancer types, ATRX is less well characterized.

The ATRX gene encodes the alpha thalassemia mental retardation X-linked syndrome protein, which is a member of the SWI/SNF DNA helicase family of chromatin remodeling proteins. Mutations in the gene are associated with a variety of cancers including adult lower-grade gliomas, pediatric glioblastoma multiforme, pediatric adrenocortical carcinoma, osteosarcoma, and neuroblastoma [10]. The ATRX protein has two highly conserved domains, the SWI/SNF domain and the ATRX-DNMT3-DNMT3L (ADD) domain [11, 12]. The SWI/SNF domain regulates chromatin remodeling, whereas the ADD domain modulates DNA methylation suppressing transcription, including in the telomeric repeat sequences, and is an essential binding partner of the death domain associated protein (DAXX), which regulates apoptosis [13–15]. When this gene mutates, it is believed that this is essential for abnormal telomere lengthening [16], giving cells with this mutation resistance to telomerase inhibitors and thus immortality [17].

A recent work has demonstrated that mutations in ATRX may promote a more aggressive and readily metastatic phenotype by altering NF-*κ*B signaling. NF-*κ*B activation induces expression of antiapoptotic genes and promotes multiple features associated with a prometastatic phenotype, such as cell growth, angiogenesis, and epithelial-mesenchymal transition [18–20]. In osteosarcoma, mutations in ATRX lead to upregulation of NF-*κ*B, extracellular matrix remodeling, increased B3 integrin expression, and more aggressive tumor cell phenotypes, including increased growth, migration, invasion, and metastasis [5]. In STS, ATRX loss is associated with increased chromosomal and mitotic instability, as well as a reduction in disease-specific survival [21]. ATRX mutation was also associated with targetable therapeutic vulnerabilities in STS, including increased sensitivity to radiation therapy and oncolytic herpesvirus [21].

The association between ATRX and a more aggressive sarcoma phenotype in both osteosarcoma and STS prompted our investigation of the prognostic relevance of an immunohistochemical assay to detect ATRX expression in STS. To accomplish this, we performed a retrospective study of sarcomas from an individual institution and their expression of ATRX was quantitatively determined. Comparison of progression-free survival, met-free survival, and overall survival between ATRX wild type and mutant STS revealed a significant difference in overall survival and disease-free survival in a group of STSs. These data suggest that ATRX loss may serve as a useful prognostic biomarker for STSs and warrants further prospective, multicenter evaluation.

## 2. Methods

### 2.1. Study Cohort

All data were collected under approval of the Duke University Institutional Review Board. A total of 128 sarcomas were collected after resection at an academic center between January 1998 and December of 2016 and were processed and stored at an affiliated biorepository. Resected tumors were evaluated by a pathologist with expertise in sarcoma and given a diagnosis, based on the histological subtype and a grade using the World Health Organization (WHO) criteria [22]. Formalin-fixed, paraffin-embedded specimens were chosen based on their quality and availability. Patient-specific data for specimens included the date of birth, grade, tumor size, mitotic rate, date of diagnosis, date of local recurrence, date of metastasis, definitive last follow-up, lymphovascular invasion, necrosis, and date of death. Patients with signs of metastasis on pathology or imaging within one month of their primary diagnosis were classified as having metastatic disease. Human colon and skin tissue were used as positive controls for all markers of interest. Tumors were excluded if (1) the tumors original biopsy date could not be ascertained from the medical record, (2) the biopsy quality was deemed unacceptable based on biorepository quality assurance protocols, or (3) the tumor did not stain appropriately for ATRX. After exclusion, we examined 112 sarcomas to determine an appropriate ATRX cut-off point. The characteristics of these tumors can be seen in Table 1. Of note, 77 of the biopsies obtained for this analysis were from the primary tumor site, 17 were from locally recurrent tumors, and 18 were from metastatic tumors.

### 2.2. Immunohistochemistry

For each specimen, a representative sample of tumor was selected by the pathologist, a biopsy was obtained, and a tissue microarray (TMA) was prepared. Sections of the TMA were cut in 3 *μ*m thickness, dewaxed in xylene, and rehydrated through graded alcohol to water. These TMA sections were then pressure-cooked in 0.1 M citrate buffer at pH 6.0 for 2 minutes before being immunostained. An ATRX antibody (Bio SB; clone BSB-108; cat# BSB-3298; 1 : 250) was applied to the TMA. Incubation without a primary antibody was the negative control. The human colon and skin were positive controls [23]. Only one TMA was created, and so each of the samples was only stained once for ATRX.

**Figure 1:**
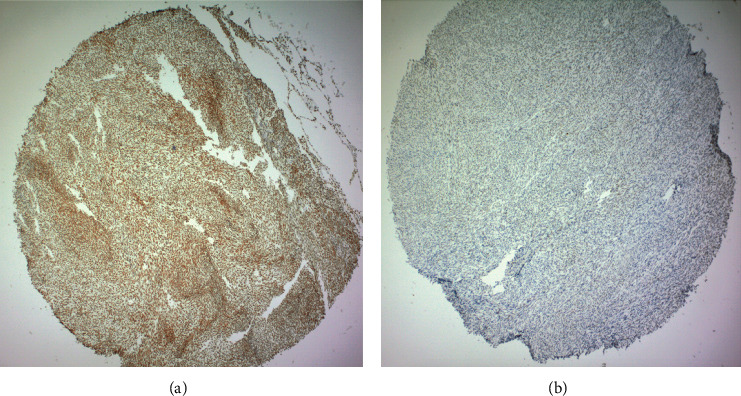
(a) Immunohistochemistry (IHC) of a sarcoma with complete expression of ATRX, which would be considered of high expression; (b) IHC of a sarcoma with no expression of ATRX, which would be considered as the low expression of ATRX.

The labelling index (LI) was utilized to determine the amount of ATRX expression inside of each tumor [24]. Typically, ATRX stains the nucleus [25].  Figure 1 shows an example of sarcomas with different levels of expression of ATRX utilizing immunohistochemistry.

The LI for each tumor in the TMA was determined by a single pathologist (DC) under a 40 times high-powered field utilizing an Olympus BX46 (Olympus Corporation, Shinjuku, Tokyo, Japan). Each microscopic image was assessed for immunoreactivity. The number of cells immunoreactive in the field, divided by the number of total cells in the field, determined the overall percentage of positive cells. In addition to the labelling index, the intensity of staining was determined qualitatively and reported as weak, moderate, and strong by DC.

High (Figure 1(a)) and low expressions (Figure 1(b)) of ATRX were found through optimization at each outcome measure. The labelling index determined which cells had a large and small amount of each marker of interest (i.e., labelling index of 50 means 50% of the cells in that sample were positive for the marker). Cut-off points (high and low) were determined utilizing the area under a receiver operating characteristic curve for ATRX. The cut-off point was determined based on the suggested threshold seen in SPSS, which determines at what point the specificity and sensitivity are equivalent and thus where the product of the sensitivity and specificity is at a maximum. This creates the largest area under the curve. It is felt that this point, statistically, is a reasonable cut-off when testing a new marker [26]. Those below the threshold were considered to have low expression of ATRX and those above or equal to the threshold were considered to have high expression of ATRX. Tumors determined to be stained correctly and that had no evidence of the antibody of interest were included in the low group.

### 2.3. Testing the ATRX Cut-Off

To test the efficacy of ATRX and its prognostic ability, we applied these to our group of primary soft tissue sarcomas. The total number of soft tissue sarcomas examined in the analysis was 68.

### 2.4. Outcome Measures

The 5-year overall survival, disease-free survival, metastasis-free survival, and local recurrence-free survival were calculated from the time of primary diagnosis—defined as the date of primary biopsy or evidence of disease on imaging—to the date of first confirmed evidence of disease progression. Metastatic disease was defined as evidence of disease progression in a site distant from the primary tumor. Locally recurrent disease was defined as a sign of disease progression in the tumor bed from which the primary sarcoma was resected or in the area adjacent to the tumor bed. Metastatic disease and locally recurrent disease were determined by the first evidence of disease on imaging. Disease-free survival was determined by the earliest sign of either metastatic or locally recurrent disease. If there were no signs of disease progression or no recorded date of death, then the end point of analysis for the patient was recorded at 5 years or the time of last follow-up, and these patients were censored at this time point. Patients who died or had any form of disease progression after the 5-year time point were censored at 5 years.

### 2.5. Statistical Analysis

Receiver operating characteristic (ROC) curves and area under the curve were utilized to determine cut-offs. Collinearity was tested among the ATRX labelling Index, tumor size, and mitotic rate with bivariate Pearson correlations. Cox regression analysis was utilized to evaluate each marker and the cell-cycle phenotypes' ability to evaluate their prognostic significance on our primary endpoints (5-year local recurrence-free survival, 5-year metastasis-free survival, 5-year disease-free survival, and 5-year overall survival). Kaplan–Meier curves were created and utilized for comparison in univariate analysis. Log-rank (Mantel-Cox) tests were utilized for comparison of each group. Multivariate analysis for each marker was performed to account for tumor size (≥5 cm vs. <5 cm) and grade (WHO classification). *T*-tests were utilized to compare age, mitotic rate, and tumor size, and goodness of fit (likelihood ratio) was utilized to compare grade. Tests were determined to be significant if the *P* value was less than or equal to 0.05. All analyses were performed in SPSS (Version 27, IBM, Armonk, NY).

## 3. Results

**Figure 2:**
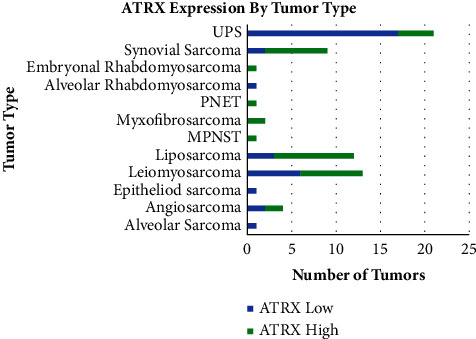
High and low expression of ATRX determined by via immunohistochemistry by tumor type for the primary soft tissue sarcoma cohort.

Based on the area under the curve from the ROC analysis, we set the labelling index cut-off to 75 for ATRX staining. The distribution of our primary soft tissue tumors that expressed high and low amounts of ATRX is shown in Figure 2.

Looking at characteristics between the two groups, the low expression group had tumors with a significantly increased mitotic rate; otherwise, there was no significant difference in the patient's age, tumors' size, or grade (Table 2).

**Figure 3:**
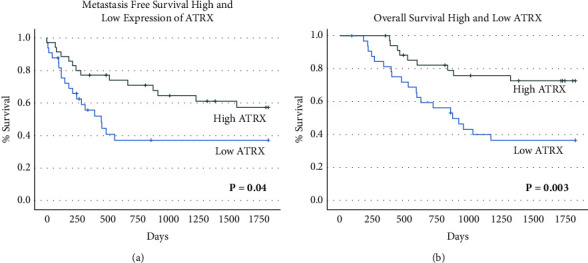
High ATRX expression correlates with overall and metastasis-free survival. (a) K-M curve of high vs. low Expression of ATRX and metastasis-free survival demonstrated a statistically significant decreased risk of metastasis in the high expression group vs. the low expression group (chi-square: 4.17, *P*=0.04). (b) K-M curve of high vs. low Expression of ATRX and overall survival that shows a statistically significant decreased risk of death in the high expression group vs. the low expression group (chi-square: 8.65, *P*=0.003).

High expression of ATRX was found to be a positive prognostic indicator for metastasis-free survival (Figure 3(a)) and overall survival (Figure 3(b)). These results persisted even after controlling for grade and tumor size (Table 3).

**Figure 4:**
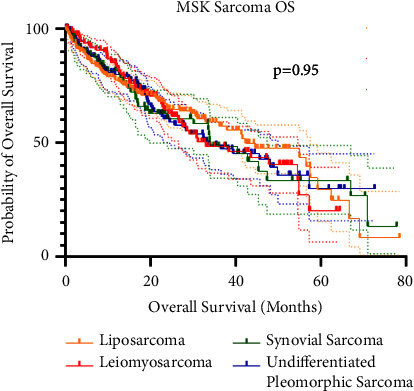
Survival curves for the top four soft tissue sarcomas in our dataset created from the data in cbioportal [27, 28]. The solid lines are the average survival of that sarcoma after diagnosis, with the dotted lines representing the 95% confidence intervals at those time points. These demonstrate that there is no difference in overall survival between these sarcomas (*P*=0.95).

Finally, utilizing the MSK large next-generation sequencing database [27–29], there was no difference in survival between the top four soft tissue sarcomas in our patient cohort (Figure 4).

## 4. Discussion

ATRX is one of the most frequently mutated genes in STS, along with TP53 and RB1 [4]. ATRX has wide-ranging cellular consequences, including a role in chromatin remodeling [30], telomere elongation [5, 12, 16, 31, 32], regulation of transcription, DNA repair, mitotic recombination [33], NF-kB signaling, and invasion [5]. Additional work has demonstrated that ATRX plays important roles in epigenetic regulation, modulating PRC2 activity [21].

The present study suggests that ATRX expression is a favorable prognostic indicator of overall survival and metastasis-free survival in patients with STS, even after controlling for tumor grade and size. Further, utilizing a larger dataset of soft tissue sarcomas (cbioportal [27, 28]), we were able to demonstrate that there is no difference in overall survival between our top four soft tissue sarcomas. This demonstrates that although we have a heterogenous group of tumors, we feel that ATRX expression and its prognostication of survival are likely broadly applicable to soft tissue sarcomas as a whole. While the exact role of ATRX in sarcomagenesis is still being investigated, its role as a prognostic indicator in the treatment of STS is more well established. While the current standard of treatment for STS is a combination of radiation and surgical resection [34], almost half of these patients will develop metastatic disease [34]. Those patients that develop metastatic disease have an estimated survival of about 20% at 5 years [2] and limited therapeutic options. As our understanding of how ATRX influences aggressivity in sarcoma continues to be developed, an important aspect of this will be to explore therapeutic vulnerabilities in patients with ATRX mutation or loss. For now, the results may give clinicians a better ability to counsel their patients in terms of their prognosis and may help determine what patients need a more aggressive approach to their treatment regimen.

## 5. Limitations

The main limitation in the present study is the relatively small size of the patient cohort (*n* = 128). Future studies could use a multi-institutional approach to increase subject numbers and explore differences by specific subtype.

## 6. Conclusion

High expression of ATRX is a positive prognostic indicator of overall survival and metastasis-free survival in patients with STS. This is consistent with studies in osteosarcoma, which indicate possible mechanisms through which loss of ATRX leads to more aggressive phenotypes. Future prospective clinical studies are required to validate the prognostic significance of these findings.

## Figures and Tables

**Table 1:** Tumor characteristics.

Sarcoma type	Size (cm)	Grade				Total
		1	2	3	NA	
Alveolar Rhabdomyosarcoma	<5				2	2
	≥5					

Angiosarcoma	<5			2	1	5
	≥5			2		

ASPS^*∗*^	<5			1		1
	≥5					

Chondrosarcoma	<5		1			6
	≥5		3	1		
	NS				1	

Clear cell sarcoma	<5					1
	≥5					

Embryonal Rhabdomyosarcoma	<5					1
	≥5					
	NS				1	

Epithelioid sarcoma	<5					1
	≥5			1		
	NS					

Ewing sarcoma	<5				1	2
	≥5			1		

Fibrosarcoma	<5		1			1
	≥5					

Leiomyosarcoma	<5	1	2			17
	≥5	1	5	7	1	

Liposarcoma	<5					16
	≥5		1	15		

MPNST^*∗*^	<5					1
	≥5			1		

Myxofibrosarcoma	<5	1				4
	≥5		1			
	DNS		1	1		

Osteosarcoma	<5		2	3		11
	≥5		1	5		
	DNS			1		

Pleomorphic Rhabdomyosarcoma	<5			1		1
	≥5					

PNET^*∗*^	<5					1
	≥5			1		

Synovial sarcoma	<5			2		12
	≥5		1	9		

UPS	<5		1	3		29
	≥5		4	21		

Total		3	24	78	7	112

^*∗*^ASPS: alveolar soft parts sarcoma, MPNST: malignant peripheral nerve sheath tumor, PNET: primitive neuro-ectodermal tumor, UPS: undifferentiated pleomorphic sarcoma.

**Table 2:** Comparison of patient characteristics of the high and low ATRX expression groups.

	Group	Mean	*P* value
Size (cm)	High expression	11.30 ± 9.24	0.34
	Low expression	13.31 ± 7.68	

Mitotic rate	High expression	11.00 ± 13.63	0.02
	Low expression	22.03 ± 21.90	

Age (years)	High expression	58.03 ± 24.38	0.52
	Low expression	61.30 ± 16.76	

Grade	Low expression	High expression	

1	0	2	LR: 4.43, *P*=0.11
2	5	9	
3	27	22	

**Table 3:** Cox regression survival analysis of high and low expression of ATRX when controlling for grade and tumor size.

ATRX							
Univariate	Hazard ratio	95% CI	*P*	Multivariate	Hazard ratio	95% CI	*P*
OS	0.33	0.15–0.72	0.005	OS	0.38	0.17–0.85	0.02
DFS	0.61	0.32–1.15	0.13	DFS	0.63	0.34–1.19	0.16
MFS	0.49	0.25–0.99	0.05	MFS	0.49	0.24–0.99	0.05
LRFS	1.30	0.47–3.57	0.62	LRFS	1.35	0.48–3.77	0.57

## Data Availability

The data used to support the findings of this study are restricted by the Duke Health institutional Review Board in order to protect patient privacy and further data are available from the corresponding author upon reasonable request.
